# Digital technology for orthognathic surgery training promotion: a randomized comparative study

**DOI:** 10.7717/peerj.13810

**Published:** 2022-08-02

**Authors:** Zhan Su, Yao Liu, Wenli Zhao, Yuanyan Bai, Nan Jiang, Songsong Zhu

**Affiliations:** State Key Laboratory of Oral Diseases, National Clinical Research Center for Oral Diseases, Department of Oral & Maxillofacial Surgery, West China Hospital of Stomatology, Sichuan University, Chengdu, China

**Keywords:** Resident training, Digital technology, Orthognathic surgery, Skill training

## Abstract

**Background:**

This study aims to investigate whether a systematic digital training system can improve the learning efficiency of residents in the first-year orthognathic surgery training course and evaluate its effectiveness in teaching orthognathic surgery.

**Methods:**

A digital training system was applied, and a comparative research approach was adopted. 24 first-year orthognathic surgery residents participated in the experiment as part of their professional skill training. The Experimental group was required to use a digital training system, and the Control group was trained in lectures without digital technologies. Three indicators, including theoretical knowledge and clinical operation, were assessed in tests, and evaluations from instructors were analyzed to evaluate learning efficiency.

**Results:**

The results showed that the scores in theoretical tests, practical operations, and teacher evaluations, the Experimental groups were all higher than the Control group (*P* = 0.002 for anatomy, *P* = 0.000 for operation theory) after using digital technology, except for the understanding of complications (*P* = 0.771). In addition, the questionnaire survey results showed that the study interest (*P* = 0.001), self-confidence (*P* = 0.001), satisfaction (*P* = 0.002), and academic performance (*P* = 0.001) of the residents of the Experimental group were higher than those of the Control group.

**Conclusions:**

The outcomes indicated that the digital training system could benefit orthognathic residents’ learning efficiency, and learning interest and teaching satisfaction will also improve.

## Introduction

Orthognathic surgery is designed to correct dentofacial deformities resulting from skeletal disharmonies (Khechoyan, 2013). The surgery process is intricate because of the multifaceted surgical anatomy and the complexity of dentofacial deformities. High accuracy of surgical operations and proficiency of the surgeon are critical for the surgical outcomes (Antonini et al., 2020; Relle & Silegy, 2004). Current training for surgeons is delivered in traditional lectures, and residents learn about anatomical knowledge and surgical operations via books, handouts, and slides combined with medical practice. However, this conventional form of education lacking three-dimensional (3D) visualization and hands-on practice may compromise residents’ learning efficiency and understanding of the topographic anatomy and complex surgical process. As a result, the efficiency of learning is low, and less than 19% of residents continue to engage in orthognathic surgery after training (Adebayo et al., 2017; Tahim et al., 2014).

Currently, digital technologies have become crucial tools and taken on significant roles in medicine, which have also been richly demonstrated in the assessment, understanding, and treatment of oral and maxillofacial disorders (Freeman et al., 2017). For example, virtual surgical planning (VSP) in orthognathic surgery facilitates diagnosis, treatment planning, and evaluation of treatment outcomes of dentofacial deformities, which has been applied in clinical practice and has achieved satisfactory outcomes (Joda et al., 2019). However, in contrast to the clinic’s wide application, the intervention of digital technologies in orthognathic surgery teaching and training is still a budding area (Berton et al., 2020; Coyne et al., 2019).

For medical education, Huffman & Ekstrom (2020) and Gumaa & Rehan Youssef (2019) indicated that residents with good ability in spatial imagination could understand the surgery concepts more thoroughly. Meanwhile, Hoyek et al. (2014) and Zhao, Patel & Cohen (2012) proved that 3D digital technologies such as 3D images of anatomy, visual surgery simulation could improve residents’ spatial imagination and understanding of surgical procedures, which is critical and meaningful for orthognathic surgery training. Some studies have explored the application of digital technology to orthognathic surgery training, particularly VR technologies (Arikatla et al., 2018; Sytek et al., 2021; Zaragoza-Siqueiros et al., 2019). For instance, a novel VR approach based on haptic technology was introduced and validated for computer-aided cephalometry. Twenty-one residents performed a range of case studies using haptic-enabled digital cephalometric analysis. They proved that by the VR technology, the errors in the cephalometric analysis had been reduced and the landmarking became more feasible and intuitive. Virtual reality has improved residents’ knowledge and proved effective in teaching clinical reasoning and patient evaluation (Medellin-Castillo et al., 2016; Sytek et al., 2021). Nevertheless, the current research still has some limitations. Firstly, previous studies have mainly compared the time and operation of simulated surgery by participants before and after training, lacking the assessment of theoretical knowledge and feedback from instructors. Secondly, few studies have focused on the critical role of learning motivation, which is essential for beginners. Moreover, other studies mainly concentrated on new applications in orthognathic surgery training, such as VR technology (Huffman & Ekstrom, 2020), and the complete digital training system has not yet been completed been established.

Therefore, this study was proposed to comprehensively assess whether digital technologies, including digital 3D images, digital model surgery, digital guide plate, and visual surgery simulation, can improve residents’ learning efficiency in the first-year orthognathic surgery training course. Through this research, we explored the best way to introduce digital technology into orthognathic surgery training, improve training programs, and evaluate the effects of this education in clinical practice.

## Methods

### The traditional orthognathic surgery training method

**Figure 1:**
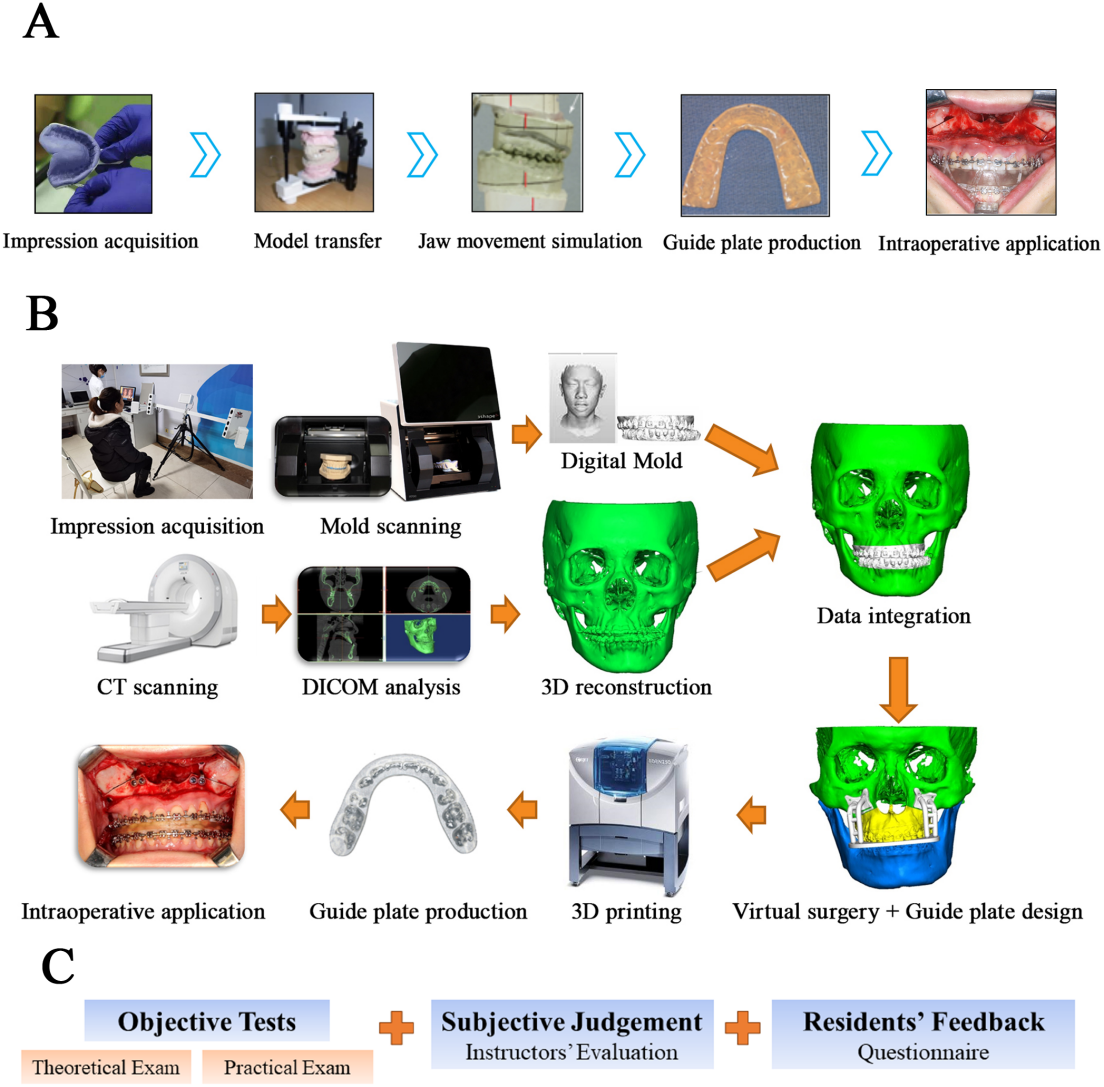
Flow chart of the traditional training method (A) , the digital technology training system (B), and the evaluation of teaching (C).

To achieve satisfactory therapeutic effects in both oral function and facial appearance, preoperative measurement and analysis and the application of surgical guide plate are of great significance in orthognathic surgery. In the traditional training method (Fig. 1A), residents are first taught by instructors in the form of lectures. Subsequently, they review the knowledge and finish their homework to obtain a deeper understanding of orthognathic surgery. Third, they complete surgery simulation with a plaster model and produce the surgical guide plate using self-curing resin. Lastly, they assist the instructors with an actual surgery to gain practical experience.

### The digital training system for orthognathic surgery

We have systematized a digital training system to test the residents’ learning efficiency assisted by digital technology, as shown in Fig. 1B. It is a standardized process consisting of three parts: data collection and integration, surgery simulation, and guide plate preparation. Briefly, first, the facial and oral anatomy information is collected by facial scanning (3dMD Modular Camera Unit Model, MCU 1−3.3) and dental mold scanning (TRIOS intraoral scanner system, T12A). The 3D images of the oral cavity can clearly reflect the details of teeth structure and dentofacial deformities of patients. At the same time, the Digital Imaging and Communications in Medicine (DICOM) data of patients can be obtained by CT scanning (Meyer CBCT, SS-X9010DPro-3DE). After the DICOM data is reconstructed in a 3D structure, they can be combined with the 3D images of the oral cavity to get a complete dentofacial mold. Second, surgeons determine the cutting line, the amount of bone to be removed, and the operation process in the simulated surgery, and the guide plate can be designed in this process. The residents can practice the surgery on the virtual maxilla-mandibular model multiple times. Third, the guide plate is established based on the simulated surgery and produced by 3D printing (Stratasys, Objet Eden 260V) for the actual application. Residents can check the guide plate during the operation and find out the inconsistencies between the simulated surgery and actual conditions. All participants could operate and design independently after training and practice.

### Research design

This study was conducted according to the guidelines set forth by the Declaration of Helsinki and approved by the Institutional Review Board of the West China Hospital of Stomatology, Sichuan University (No. WCHS-IRB-CT-2019-221). Written informed consent was obtained from all participants. The study was based on the orthognathic residents’ training course set up by the Department of Oral & Maxillofacial Surgery, West China Hospital of Stomatology, Sichuan University. A total of 24 first-year orthognathic residents participated in the study as part of their professional skill training. A randomized control trial was performed to obtain an average grouping. The random setting is as follows: a total of 34 first-year orthognathic residents were divided into two groups (male/female) firstly according to gender. Then 12 residents were randomly selected in each of two packets using a random-number table consisting of 24 participants ultimately, which can draw random sampling scientifically (Zhang et al., 2020). We randomly assigned participants into two groups (*n* = 12): one group was trained by the traditional orthognathic surgery training method (Control group), and another group was trained with the digital training system (Experimental group). The age distribution of the participants was between 23–27 years. None of the participants had received orthognathic surgery courses and had no exposure to the orthognathic surgery before this study. The experiment started at the beginning of the semester, lasted for one year, and was evaluated at the end of the semester. The total class hours are the same for all participants. Each lesson was 2 hours long once a week and taught by instructors with more than 10 years of teaching experience. The theoretical content covered preoperative preparation, basic surgery principles, applied anatomy, rigid internal fixation, and operative procedure, and is based on *Orthognathic Surgery* (People’s Health Publishing House).

To evaluate the learning efficiency of residents through the traditional and digital training system, two objective tests (the theoretical exam and practical exam) and one subjective judgement (instructors’ evaluation of residents’ performance) were included in the analysis. The whole evaluation process is shown in Fig. 1C. Test 1 was in the form of a paper exam. The instructors prepared questions based on three sections: the basic knowledge of anatomy, complications, and surgical points involved in the teaching. Including 20 multiple-choice questions, 20 blank filling questions, and 5 short answer questions, with a total score of 100. We prepared the questions refer to *the Standardized Training and Assessment Outline for Residents of Oral and Maxillofacial Surgery* (National Health Commission of the PRC) and *Accreditation Standards for Advanced Dental Education Programs in Oral and Maxillofacial Surgery* (American Dental Association).

Test 2 was a practical exam conducted on imitation head mold. The content of the exam is the LeFort I osteotomy for maxillary with sagittal split ramus osteotomy for mandibular on the bony Class III malocclusion imitation head mold. The combined use of the two surgical approaches is the most common procedure in clinical practice. Bimaxillary surgery can better examine the residents’ understanding of the relationship between the spatial anatomical structure of the jaw. Test 2 is divided into five items: design of surgical incisions, design of osteotomy lines, use of guide plate, placement of bone blocks, and fixation of bone blocks. Each item’s operation, presentation, and time consumption were examined, and each item was scored from 0 to 20 out of 100 points. Since the two groups of residents learned different methods of making the guide plate, we used a uniform guide plate in the operation test, and the test mainly examined the residents’ understanding and proficiency of the main points of the operation. After completing the exams, the scores were calculated, and the two groups’ results were compared.

Simultaneously, three instructors with more than ten years of teaching and clinical experience who were not involved in this teaching activity gave subjective evaluations by observing the whole process of residents’ practical examinations and asking random questions. Prior to participating in the evaluation, the instructors developed uniform scoring criteria referred to *the Detailed Rules for Assessment and Scoring of Standardized Training for Residents* (National Health Commission of the PRC). In their marks, A, B, C, and D represented excellent, good, general, and bad, respectively.

### Questionnaire

To collect residents’ feedback about two different learning systems, a short questionnaire consisting of ten questions was distributed to all residents in Control group and Experimental group at the end of the course. The questionnaire contains 10 questions. Each question used a 5-point Likert scale, in which 1 represents “strong disagreement” and 5 represents “strong agreement”. Likert scale is a close-ended, forced-choice scale used in a questionnaire that provides a series of answers that go from one extreme to another. Likert scales are widely used in medical education research (Horan, Murphy & O’Brien, 2020; Manalo et al., 2021; Yang et al., 2021). As shown in Table 1, each question had a particular focus. Q1 and Q10 assessed respondents’ satisfaction; Q2 and Q9 assessed learning interest; Q3 and Q4 assessed academic outcomes; Q5 and Q8 assessed respondents’ confidence for engaging in orthognathic surgery; and Q6 and Q7 assessed the ability of handling practical operation. These aspects can be used as direct or indirect indicators of teaching effect evaluation (Alamrani et al., 2018; Kouz et al., 2020).

**Table 1:** Questionnaire given to all participants.

**Question**	**Score**				
1. I like this way of learning.	1	2	3	4	5
2. My attention was quickly and easily caught by orthognathic surgery.	1	2	3	4	5
3. Based on the acquired teaching content and my own understanding, I can clearly understand the anatomical structure of the surgical area, the adjacent important nerves and blood vessels in my mind.	1	2	3	4	5
4. This teaching method can effectively help me understand orthognathic surgery procedures and deepen my understanding of orthognathic surgery.	1	2	3	4	5
5. Based on the teaching content and my own understanding, I can independently carry out the orthognathic surgical design in the future.	1	2	3	4	5
6. The guide plate preparation is not difficult for me at all.	1	2	3	4	5
7. I can complete the role of the first assistant in surgery very well.	1	2	3	4	5
8. I have the confidence to know what I should learn in the course.	1	2	3	4	5
9. I didn’t feel bored at the end of the course.	1	2	3	4	5
10. I am satisfied with the teaching method I have experienced	1	2	3	4	5

### Data analysis

SPSS 26.0 Statistics was used to do data analysis. Independent-samples T-tests were conducted and analyzed to determine statistically significant differences between the Experimental and Control groups of scores of three sections related to anatomy, operation theory, and complication in Test1 and scores of practical operations in Test2. Non-parametric tests Mann–Whitney U and Wilcoxon rank-sum test were performed to analyze the qualitative data obtained from subjective evaluation and the questionnaire. The significance level was set as *p* < 0.05.

## Results

### Theoretical tests

The scores of the theoretical test were collected separately and analyzed by *t*-test to investigate the difference between the Control group and the Experimental group as demonstrated in Table 2. For orthognathic surgery related anatomy, residents’ scores were 73.72 ± 6.05 and 82.33 ± 5.45 in the Control group and the Experimental group, respectively, and the scores of operation theory were 70.67 ± 6.37 and 83.42 ± 7.18, respectively. The results of the *t*-test indicated that residents of the Experimental group mastered the knowledge of anatomy and operation much better (*P* = 0.002 for anatomy, *P* = 0.000 for operation theory). Regarding complication understanding, residents’ scores were 78.42 ± 5.73 and 77.75 ± 5.34 in the Control group and the Experimental group, respectively, where *P* = 0.771 indicated that no statistically significant difference was found between the two groups.

**Table 2:** The scores of the theoretical test.

**Test**	**Group**	**Score**	**F**	**t**	**P**
anatomy	Control	73.72 ± 6.05	0.179	−3.580	0.002
	Experimental	82.33 ± 5.45			
operation theory	Control	70.67 ± 6.37	0.187	−4.601	0.000
	Experimental	83.42 ± 7.18			
complication	Control	78.42 ± 5.73	0.189	0.295	0.771
	Experimental	77.75 ± 5.34			

### The test on the practical operation and subjective judgments from instructors

As shown in Table 3, the test scores on the practical operation were 70.83 ± 4.63 and 85.67 ± 5.00 for the Control group and the Experimental group, respectively, with a maximum score of 94 in Experimental group. The statistical analysis showed that significant differences existed (*P* = 0.001) between the Control group and the Experimental group on the practical operation, implying residents from Experimental group could perform better in the clinical practice. Meanwhile, the results of feedback from instructors were summarized in Table 4. Significant differences also existed between the two groups (*P* = 0.013), indicating the instructors gave more recognition to the Experimental group’s practical operation than the Control group, which was in accordance with the test result on the practical operation.

### Questionnaire

A questionnaire containing 10 questions was distributed to both groups of residents anonymously. The descriptive statistics of the questionnaire was shown in Table 5. The mean values of each evaluated factor provided by the Control group residents, who experienced conventional training, were lower than those provided by the Experimental group residents, who experienced the digital training. In particular, the difference in mean score for Learning Interest between the two groups was 12, indicating that the Experimental group enjoyed the learning process much more than the Control group (*P* = 0.001). Meanwhile, residents in the Experimental group could understand the anatomical structure of the surgical area and orthognathic surgery procedures better, as shown in Academic Outcome (*P* = 0.001), which was coherent with the outcome from theoretical tests. For the operation part, residents in the Experimental group had much more confidence in clinical work such as guide plate preparation or first assistant duty (*P* = 0.001), which is consistent with the previous test result on the practical operation and subjective judgments from instructors. Therefore, residents in the Experimental group were more satisfied with the training (*P* = 0.002) and held more confidence (*P* = 0.001) in the clinical practice than those in Control group.

**Table 3:** The scores of the test on the practical operation.

**Group**	**Score**	**F**	**t**	**P**
Control	70.83 ± 4.63	0.013	−7.544	0.001
Experimental	85.67 ± 5.00			

**Table 4:** The results of feedback from instructors.

**Group**	**Frequency**					
	**Level A**	**Level B**	**Level C**	**n**	**Z**	**P**
Control	2	5	5	12	2.495	0.013
Experimental	8	4	0	12		

**Table 5:** The descriptive statistics of the questionnaire.

**Question**	**Factor**	**Group**	**Rank mean**	**Z**	**P**
2 & 9	Learning Interest	Control	6.50	4.210	0.001
		Experimental	18.50		
6 & 7	Practical operation	Control	7.21	3.731	0.001
		Experimental	17.79		
3 & 4	Academic Outcome	Control	7.79	3.335	0.001
		Experimental	17.21		
5 & 8	Confidence	Control	7.13	3.832	0.001
		Experimental	17.88		
1 & 10	Satisfaction	Control	8.25	3.007	0.002
		Experimental	16.75		

## Discussion

We have designed a complete training system that incorporates digital technology, allowing residents to participate in impression acquisition, model scanning, 3D reconstruction, 3D printing up to simulation model manipulation, simulating the complete clinical experience. This immersive teaching method is more vivid than the traditional one, and the results confirmed a higher level of resident acceptance. To comprehensively evaluate the digital training system’s efficacy, three theoretical exams, one operation test, subjective judgments, and one questionnaire were conducted in this study. This evaluation method covers multiple dimensions of “objective-subjective”, “residents-instructors”, and “theory-operation”, which can more comprehensively evaluate the strengths and weaknesses of both training systems. The results showed the improvement of residents’ learning efficiency brought by digital training system when compared to traditional training method, except for the complication part. This may be because the understanding of complications involves multiple disciplines, such as cardiovascular, neurological, and anesthesia. It may be difficult to cover so many fields in the limited time available for training in orthognathic surgery. These outcomes are in accordance with the conclusion from other surgical subjects, such as orthopedics, otolaryngology, and neurosurgery (Andersen et al., 2016; Walbron et al., 2020; Zawy Alsofy, Sakellaropoulou & Stroop, 2020).

The improvement is speculated to be proportional to the difficulty of the surgery process (Clarke, 2021; Yeung et al., 2021). Orthognathic surgery involves a series of complex anatomical structures such as the maxilla and mandible, inferior alveolar neurovascular, facial nerve, internal maxillary artery, facial artery, mental nerve, temporomandibular joint, etc (Copson et al., 2021). Traditional lectures in which flat images and texts are used for illustration cannot fully reflect the 3D spatial relationship of the above structures. Spiral CT can provide 3D images of the jaws, but even with the help of spiral CT, residents still need good spatial imagination and clinical experience to appreciate the exact anatomy of the surgery area. The application of digital technologies can clearly show the spatial position relationship of the above structures. Students can use software to move, zoom arbitrarily, and rotate the 3D image of the reconstructed anatomical structure to visually observe the complex anatomical structures (Iwanaga et al., 2021; Karanxha et al., 2021). The resident can view the anatomy of the upper and lower jaws from all angles, simulate the various osteotomy lines and the critical structures that may be damaged by the various osteotomy lines, and move the osteotomy block in a 3D direction. This will help residents understand the operation more intuitively and upgrade their surgical coordination ability (Akhtar et al., 2015). Moreover, after the postoperative explanation by instructors, residents can further study the operation and match the actual surgical process by themselves, which can yield a good training effect.

Typically, the stronger the motivation for learning, the more time and energy students will devote to learning, and the easier it is to achieve the purpose of efficient learning and teaching (Fidan & Tuncel, 2019). Compared with other medical courses, orthognathic surgery has unfavorable factors such as difficulty remembering the terminology, tedious learning process, and high spatial comprehension requirements, which are not conducive to learning and teaching. Therefore, the study of orthognathic surgery requires residents to have a strong learning motivation. All the residents in this study are beginners, therefore, arousing their learning enthusiasm and building their self-confidence is very important. Our study suggests that Experimental group residents showed more interest and confidence after the training than Control group. It is reported that the improvements of interest and confidence resulting from the good interactive experience provided by the digital training system (Fidan & Tuncel, 2019; Sahin & Yilmaz, 2019; Yip et al., 2018). Due to the complexity of facial anatomy and poor surgical view, orthognathic surgery is complicated to demonstrate to beginners, especially those with poor spatial imagination, through flat learning materials such as books, handouts, and PowerPoints. With the assistance of digital technologies, the residents can experience being guided in a “real” operation. In this way, the dimensions of instructions are upgraded. Moreover, Experimental group residents feel more satisfied with the training process, which should be a matter of course for these highly interested and confident trainees.

This study found that applying systematic digital technology in orthognathic surgery training is a good beginning. However, it also has limitations. The primary limitation of this study is the inadequate sample size of residents: there were only 12 residents in one group. Thus, future studies with increased sample size will be needed. Another aspect is the lack of long-term evaluation of residents. Although one year maybe sufficient for residents’ training, long-term clinical experience is equally important for the career development of residents. It is hard to predict the long-term effect of the two groups. We believe that digital technology has a profound impact on Experimental group’s residents and can have a positive impact in later clinic practice as well. Therefore, in order to get a comprehensively evaluation as possible, various assessments were applied in this study, which can technically provide a stable conclusion (Chen & Lu, 2022; Ghedin & Aquario, 2008). Thirdly, the interactive experience design and the operational logic can be improved. Some studies have confirmed that auditory and haptic senses can stimulate learners’ interest in learning, improve concentration and creativity, and contribute to the implementation of medical teaching (Breese et al., 2020; Choi et al., 2021; Rodman & Trivedi, 2020; Wu et al., 2018; Yi et al., 2020). Therefore, it is possible to provide residents with a better operating experience through hardware and software upgrades.

## Conclusions

Our findings suggest that the systematic digital training system can significantly improve the residents’ learning efficiency in the first-year orthognathic surgery training course. In the coming years, it would be meaningful to enlarge the sample size, upgrade the digital interactive devices and retest these residents after their orthopedic residency to assess their future performance and how it changes with time. Digital training system should be widely applied in medical training. It is hoped that this paper can provide a positive reference for relevant research areas.

##  Supplemental Information

10.7717/peerj.13810/supp-1 Supplemental Information 1 Raw data: results of feedback from instructors

10.7717/peerj.13810/supp-2 Supplemental Information 2 Raw data: descriptive statistics of the questionnaire

10.7717/peerj.13810/supp-3 Supplemental Information 3 Raw data: The scores of the test on the practical operation

10.7717/peerj.13810/supp-4 Supplemental Information 4 Raw data: The scores of the theoretical test

## Additional Information and Declarations

### Data Availability

The following information was supplied regarding data availability: The raw data are available in the Supplemental Files.
